# μ-Methyl­ene-bis­[di­bromido(diethyl ether-κ*O*)aluminium(III)]: crystal structure and chemical exchange in solution

**DOI:** 10.1107/S2056989021005302

**Published:** 2021-05-21

**Authors:** Ray J. Butcher, Andrew P. Purdy

**Affiliations:** a Department of Chemistry, Howard University, 525 College Street NW, Washington DC 20059, USA; b Chemistry Division, Code 6123, Naval Research Laboratory, 4555 Overlook Av, SW, Washington DC 20375-5342, USA

**Keywords:** crystal structure, methyl­ene bridged aluminum, aluminum diethyl ether complex, solution NMR studies

## Abstract

In the title compound, [Al_2_Br_4_(CH_2_)(C_4_H_10_O)_2_], the mol­ecule lies on a crystallographic twofold axis passing through the bridging C atom. Each Al^III^ atom is four-coordinate, being bonded to two bromide ions, bridging the CH_2_ group as well as the oxygen atom of a diethyl ether ligand in a slightly distorted tetra­hedral arrangement with angles ranging from 101.52 (8) to 116.44 (5)°. The Al—CH_2_—Al angle, 118.4 (2)°, is the smallest observed for a structure where this moiety is not part of a ring. In the crystal, weak C—H⋯Br inter­actions, characterized as *R* ^2^_2_(12) rings, link the mol­ecules into ribbons in the [101] direction. The title compound is monomeric and coordinatively saturated in the solid state, as each aluminum is four-coordinate, but in solution the ether mol­ecules from either or both Al atoms can dissociate, and would be expected to rapidly exchange, and this is supported by NMR data.

## Abstract 2

The crystal structure of μ-methyl­ene-bis­[(di­bromo)(diethyl ether-κ*O*)aluminium(III)] has established that the Al—CH_2_—Al angle, 118.4 (2)°, is the smallest observed for structure where this moiety is not part of a ring.

## Chemical context   

There is great current inter­est in the chemistry of reduced aluminum (Klemp *et al.*, 2001 ▸, Bonyhady *et al.*, 2018 ▸) and aluminum carbon (carbaalanes) clusters (Stasch *et al.*, 2002 ▸; Uhl & Roesky, 2002 ▸; Kumar *et al.*, 2004 ▸) as well as aluminum–carbon nanoparticles (Diaz-Droguett *et al.*, 2020 ▸) because of their inter­esting structural chemistry and many theoretical studies have been carried out on potential derivatives and as analogs of the better known boron examples (Attia *et al.*, 2017 ▸). This has lead to a renaissance in the chemistry of aluminum (Roesky, 2004 ▸). In view of this chemistry, there is a need for easily prepared precursors for the synthesis of these reduced aluminum and carbaalane clusters, and this is the motivation behind preparing organometallics with two or more Al atoms on a carbon atom. The synthesis of methyl­ene bis­(aluminum halides) has been described before (Ort & Mottus, 1973 ▸; Lehmkuhl & Schäfer, 1966 ▸).
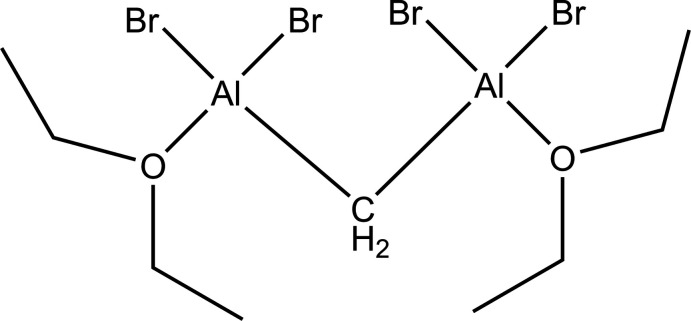


## Structural commentary   

**Figure 1:**
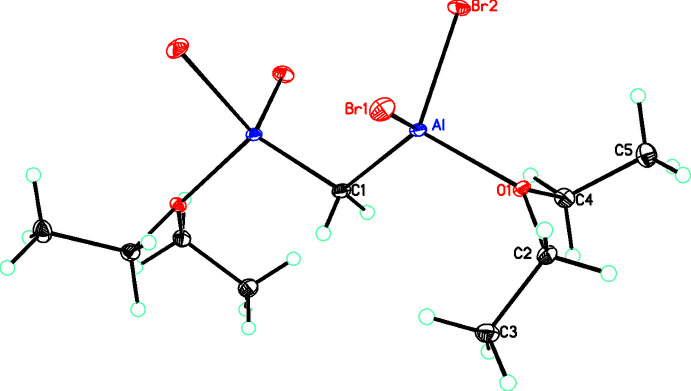
Mol­ecular diagram showing the atom labeling (symmetry operation to generate the complete mol­ecule, −*x*, *y*, 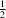 − *z*). Atomic displacement parameters are at the 30% level.

In the structure of the title compound, [Al_2_(C_9_H_22_Br_4_O_2_)] (**1**), the mol­ecule lies on a crystallographic twofold axis passing through C1 (see Fig. 1 ▸). Each Al atom is four-coordinate, being bonded to two bromide ions and the bridging CH_2_ group as well as the oxygen of a diethyl ether ligand in a slightly distorted tetra­hedral arrangement (τ_4_ = 0.907; Okuniewski *et al.*, 2015 ▸) with angles ranging from 101.52 (8) to 116.44 (5)° (see Table 1 ▸). In the literature there are eight structures containing an AlBr_2_ fragment coordinated to a diethyl ether ligand (LOCMEY, Yanagisawa *et al.*, 2018 ▸; NOJYIW, Lips *et al.*, 2014 ▸; QQQGXV, QQQGYA, Semenenko *et al.*, 1973 ▸; RABCOM, Wehmschulte *et al.*, 1996 ▸; TEXNIV, Agou *et al.*, 2012 ▸; YANKON, Petrie *et al.*, 1993 ▸; YERLUD, Quillian *et al.*, 2006 ▸). In each of these structures, there is both a longer and shorter Al—Br bond distance [average Al—Br distances of 2.315 (18) and 2.30 (2) Å] with an average Al—O distance of 1.874 (14) Å. The comparable distances in **1** are 2.3046 (10), 2.3029 (9) and 1.881 (2) Å.

As indicated below, there are many instances of structures containing the Al–CH_2_–Al fragment but only one which combines this fragment along with aluminum–halogen bonding (Uhl & Layh, 1991 ▸). In this structure {[(Me_3_Si)_2_CHAlCl]_2_CH_2_}_2_, this moiety is not isolated but part of a ring in an adamantanoid cage, which would influence both its bond lengths and angles. However, there are ten instances (BELLAH, BELLEL, BELLIP, BELLOW, BELLUP (Uhl *et al.*, 2012*a* ▸); JEZFID (Layh & Uhl, 1990 ▸); JUWMOD (Uhl *et al.*, 1993 ▸); PENSEI (Uhl *et al.*, 2012*b* ▸); WOZJUQ, WOZKAX (Knabel *et al.*, 2002 ▸) where the metrical parameters of the Al–CH_2_–Al fragment are not influenced by being part of a ring. In these structures, apart from JEZFID (Layh & Uhl, 1990 ▸) and WOZJUQ (Knabel *et al.*, 2002 ▸), there are two independent Al—C bond lengths, which average 2.003 and 1.922 Å, with an overall average Al—C—Al bond angle of 132.5°. As a result of the unconstrained nature of this angle, it varies over a wide range from 126.3 to 144.4° and the value depends on the steric bulk of the Al substituents. In the smallest value in the list [BELLOV, 126.29 (13)°; Uhl *et al.*, 2012*a* ▸], the substituents attached to Al are (tri­methyl­sil­yl)meth­yl moieties, while the largest [JUWMOD, 144.4 (2)°; Uhl *et al.*, 1993 ▸] has a neopentyl as well as two [bis­(tri­methyl­sil­yl)meth­yl] groups attached. There are two structures, QQQGXV and QQQGYA (Semenenko *et al.*, 1973 ▸), which only have Br_3_ and Br_2_H as substituents on the Al, but the angles cannot be calculated since the coordinates are not available. In **1**, which lacks this steric bulk and where atom C1 lies on a crystallographic twofold axis, these values are 1.927 (2) Å and 118.4 (2)°, respectively. This latter value reflects this lack of steric bulk in the groups attached to the Al atoms.

## Supra­molecular features   

**Figure 2:**
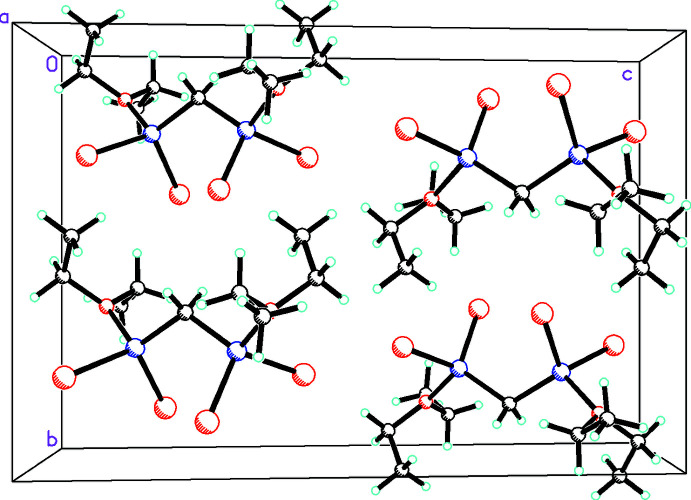
Packing diagram of **1** viewed from the [010] direction.

**Figure 3:**
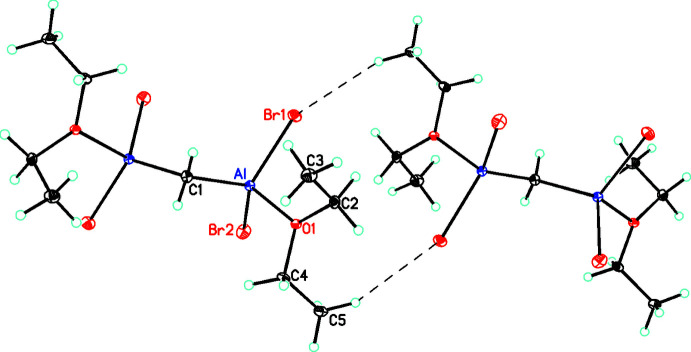
Diagram showing the C—H⋯Br inter­actions (as dashed lines) that link the mol­ecules into ribbons *via* the formation of 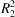
(12) rings (symmetry operation, 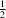 − *x*, 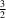 − *y*, 1 − *z*).

**Figure 4:**
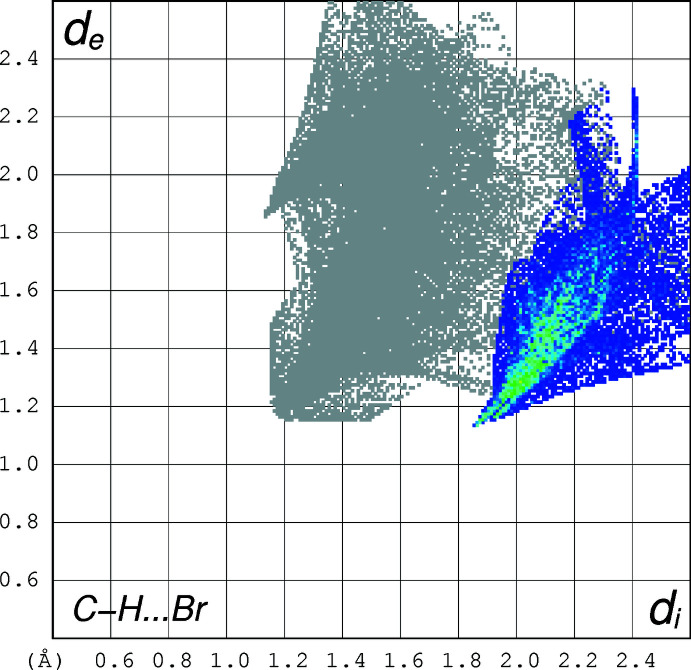
Hirshfeld surface plot highlighting the C—H⋯Br inter­actions, which make up 52.6% of all inter­actions.

As shown in Fig. 2 ▸, there are weak C—H⋯Br inter­actions, which link the mol­ecules into ribbons in the [101] direction (see Table 2 ▸). In graph-set notation (Etter *et al.*, 1990 ▸), these inter­actions can be characterized as 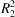
(12) rings and this is shown in Fig. 3 ▸. These inter­actions can be highlighted in a Hirshfeld fingerprint plot as shown in Fig. 4 ▸ (Spackman & Jayatilaka, 2009 ▸), which shows these features. If this is expanded to take inter­actions beyond the van der Waals radii sum cutoff (Desiraju & Steiner, 1999 ▸; Desiraju, 2011*a* ▸,*b* ▸), this plot indicates that these weak C—H⋯Br inter­actions dominate the packing and make up 52.6% of all inter­molecular inter­actions.

## Database survey   

A search of the Cambridge Structural Database [CSD version 5.41 (November 2019); Groom *et al.*, 2016 ▸] for fragments based on the structure of **1** revealed there are eight structures in the literature containing an AlBr_2_ fragment coordinated to a diethyl ether ligand (LOCMEY, Yanagisawa *et al.*, 2018 ▸; NOJYIW, Lips *et al.*, 2014 ▸; QQQGXV, QQQGYA, Semenenko *et al.*, 1973 ▸; RABCOM, Wehmschulte *et al.*, 1996 ▸; TEXNIV, Agou *et al.*, 2012 ▸; YANKON, Petrie *et al.*, 1993 ▸; YERLUD, Quillian *et al.*, 2006 ▸). There were 99 examples containing the Al–CH_2_–Al fragment, of which there are ten instances (BELLAH, BELLEL, BELLIP, BELLOW, BELLUP (Uhl *et al.*, 2012*a* ▸); JEZFID (Layh & Uhl, 1990 ▸); JUWMOD (Uhl *et al.*, 1993 ▸); PENSEI (Uhl *et al.*, 2012*b* ▸); WOZJUQ, WOZKAX (Knabel *et al.*, 2002 ▸) where the metrical parameters of the Al–CH_2_–Al fragment are not influenced by being part of a ring.

## Synthesis and crystallization   

**Figure 5:**
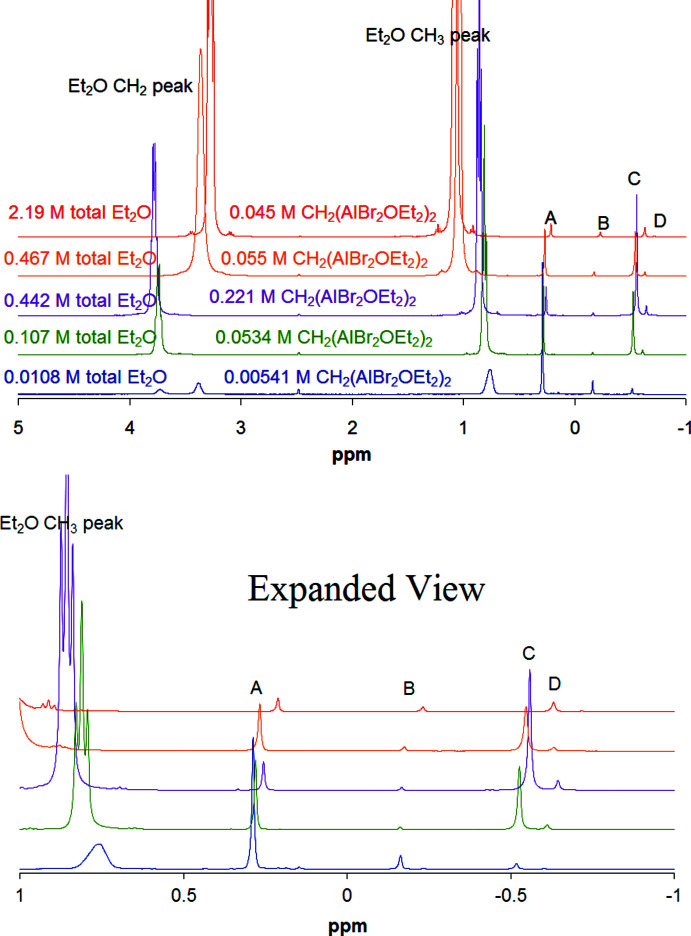
^1^H NMR spectra of the title compound in C_6_D_6_ at three different concentrations (bottom three spectra), and at an inter­mediate concentration with added ether (top two spectra). The CH_2_ group attached to Al has peaks *A*, *B*, *C*, and *D*, which are concentration dependent, and an expanded view from δ 1 to −1 ppm is shown in the lower part of the figure. The concentration of the small peak at 2.5 ppm (probably OH) is invariant in all samples and is undoubtedly due to hydrolysis caused by the release of a small amount of water during flame sealing of the NMR tubes.

Aluminum wire, cut into small pieces (3.19 g), was added slowly over several days to a stirred, dry CH_2_Br_2_ (50 mL) under N_2_ by inserting the wire through a hole in a rubber septum. After the aluminum had reacted, the mixture was filtered inside an N_2_ flow dry box and the solids were collected and pumped dry. A total of 20.22 g (96% based on Al) was isolated. A portion of this white solid was dissolved in Et_2_O and allowed to slowly evaporate inside the dry box to produce crystals of the title compound. IR (neat smeared on KBr plates, cm^−1^): [3002.90, 2982.63, 2871.84, 2964.61, 2935.43, 2920.37, 2850.50] (*m*, C—H str), 2213.10 (*w*), 1635.50 (*w*), 1463.47 (*m*), 1442.72 (*m*), 1390.14 (*s*), 1326.09 (*m*), 1281.45 (*w*), 1260.61 (*m*), 1189.28 (*m*), 1146.67 (*m*), 1088.57 (*m*), 999.64 (*s*), 985.03 (*s*), 904.06 (*w*), 879.11 (*s*), 827.67 (*m*), 796.06 (*w*), 763.57 (*s*), 723.29 (*m*), 606.38 (*s*), 545.18 (*s*), 530.16 (*s*), 463.05 (*w*). The NMR solvents were dried from sodium–potassium alloy. NMR spectra were recorded in C_6_D_6_ solution in flame-sealed tubes and were found to be concentration dependent. Proton spectra were recorded at 400 MHz on three different concentrations, and two samples of the inter­mediate concentration with added ether, and are displayed in Fig. 5 ▸. ^13^C spectrum (C_6_D_6_, 100 MHz): δ 1.34 (CH_2_, sharp), −1.46 ppm (CH_2_, broad, HHLW ≃ 150 Hz). ^27^Al spectrum (C_6_D_6_, 104 MHz): δ 93 (sharp), 132 ppm (broad, HHLW ≃ 4000 Hz).

**Safety Note:**

***This reaction should be carried out with caution as when finely divided Al flakes were used instead of Al wire, an explosion occurred.***

## Chemical exchange in solution   

The title compound (**1**) is monomeric and coordinatively saturated in the solid state, as each aluminum is four-coord­inate, but in solution, the ether mol­ecules from either or both Al atoms can dissociate and would be expected to exchange rapidly. Once an ether mol­ecule dissociates, the aluminum atom can regain four-coordination by association to a bromine atom from the other half of the same or another mol­ecule. In the C_6_D_6_ solution, there are four main proton NMR peaks visible for the CH_2_ moieties on aluminum, as shown in Fig. 5 ▸, and those peaks are labeled *A*, *B*, *C*, and *D*. Both the relative amounts and chemical shifts of peaks *A*–*D* are concentration dependent. Additionally, the NMR peaks for the ether moieties are dependent on concentration as well, as is most obvious at the lowest concentration (where the ether CH_2_ peak splits), and adding additional ether to the solution does affect the spectra, as also shown in Fig. 5 ▸. The unsolvated parent compound, CH_2_(AlBr_2_)_2_, has extremely low solubility in non-coordinating solvents such as C_6_D_6_, as one would expect if it is polymeric. While the structures of unsolvated compounds of this type are unknown, association through Al_2_Br_2_ rings is common, and this compound can easily form such rings on each end linking into an extended structure.

**Figure 6:**
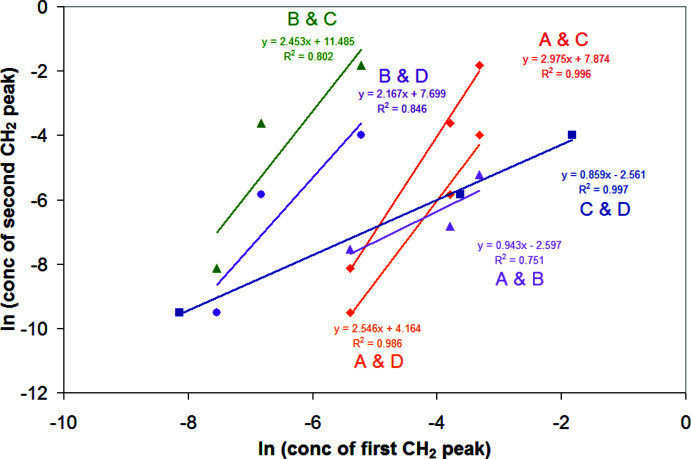
ln–ln plots of the concentrations of the mol­ecules represented by the CH_2_—Al peaks in the proton spectra. The concentration is calculated from the integral fraction of those CH_2_ resonances multiplied by the total concentration of CH_2_(AlBr_2_OEt_2_)_2_ dissolved. The slope of the line is the ratio of the degree of association of the species in solution.

**Figure 7:**
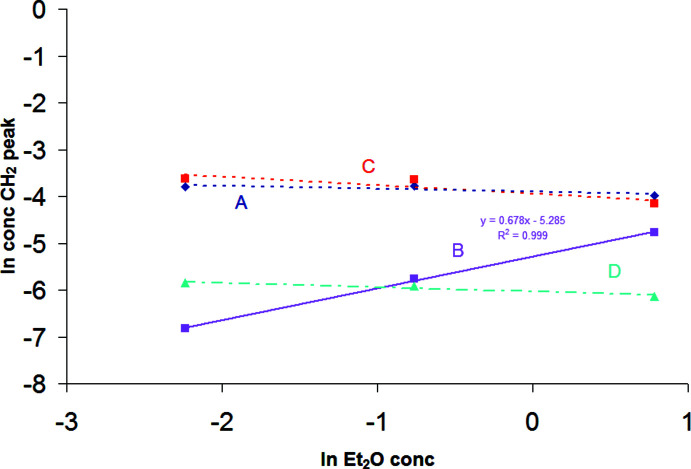
ln–ln plots of the total ether concentration on the *x*-axis and the concentration of the species represented by the CH_2_—Al peaks on the *y*-axis for the three samples with approximately equal total concentration of CH_2_(AlBr_2_OEt_2_)_2_. The relative amount of the species with peak *B* increases with ether concentration, while the other peaks decrease.

**Figure 8:**
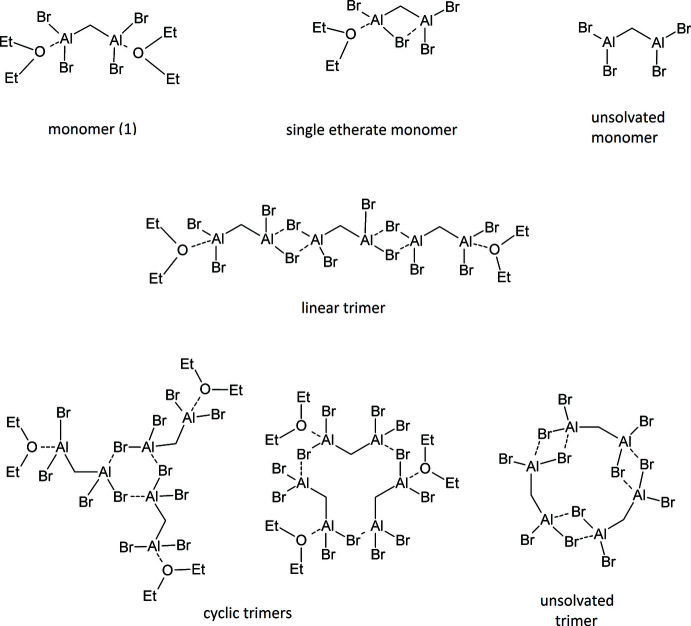
Drawings of some possible structures of monomeric and trimeric aggregates of **1**, with varying degrees of ether coordination. The possibilities exhibited here are not exhaustive.

One can determine some information as to the identity of the peaks from the concentration dependence of the spectra. If there is an exchange process between different degrees of association (for example between monomer and trimer), the ratio of oligomerization between the species can be determined by the slope of a ln–ln plot of the molar concentrations represented by each NMR peak (Purdy *et al.*, 1987 ▸). Fig. 6 ▸ shows a natural ln–ln plot for the integral fraction of the CH_2_ NMR peaks multiplied by the absolute concentration of the title compound in solution, for all six binary combinations of peaks *A*–*D*, with the linear equation between the points displayed on the chart. All combinations involving only peaks *A*, *C*, and *D* have *R* ^2^ factors near 1, showing a high linear correlation. The species with the NMR peak *C* clearly has three times the degree of association of *A*, and *D* has 2.5 times the degree of association as *A*. However, all combinations involving peak *B* with *A*, *C*, or *D* do not have as good a linear correlation, but do show that and *A* and *B* have approximately the same degree of association. Fig. 7 ▸ displays a ln–ln plot for the concentrations of peaks *A*–*D* against the ether concentration for three solutions of approximately the same concentration of the title compound with varying amounts of ether. Clearly, peak *B* correlates positively to the ether concentration, and the relative amounts of peaks *A*, *C*, and *D* have a slightly negative correlation to the concentration of ether. Therefore, we conclude that *B* is for a solution species that is more coordinatively saturated by ether than *A*, *C*, or *D*, and is probably the title compound. *A* is probably formed by the dissociation of a single ether mol­ecule, *C* is its trimer, and *D* is a partially associated trimer. Fig. 8 ▸ illustrates some possible structures of monomeric and trimeric species, with varying degrees of ether solvation, although the drawings of trimers do not exhaust the possible structures that may exist. The NMR data do not allow definite structural conclusions to be drawn for the trimers. While substantial precedent exists for compounds associated through Al_2_Br_2_ rings, and an example exists for a four-membered Al–CH_2_–Al–Br ring (PENSOS; Uhl *et al.* 2012*a* ▸), six- and eight-membered aluminum–halogen (Al*X*)_*n*_ rings are mostly known for *X*=F, although a structurally constrained Cl example does exist (GOTNEI; Tschinkl *et al.* 1999 ▸). An example exists for a linearly associated –CH_2_–AlBr_3_–AlBr_3_ moiety (KIXBEA; Ménard *et al.* 2013 ▸), which opens the possibility that a partially associated trimer could have a single dative bond in place of Al_2_Br_2_ or Al_3_Br_3_ rings.

## Refinement   

Crystal data, data collection and structure refinement details are summarized in Table 3 ▸. For the CH_2_ bridging group, the H-atom position was refined isotropically while the other H atoms were refined in idealized positions using a riding model with atomic displacement parameters of *U*_iso_(H) = 1.2*U*_eq_(C) [1.5*U*_eq_(C) for CH_3_], with C—H distances ranging from 0.98 to 0.99 Å.

## Supplementary Material

Crystal structure: contains datablock(s) I. DOI: 10.1107/S2056989021005302/jq2003sup1.cif

Structure factors: contains datablock(s) I. DOI: 10.1107/S2056989021005302/jq2003Isup2.hkl

CCDC reference: 2084539

Additional supporting information:  crystallographic information; 3D view; checkCIF report

## Figures and Tables

**Table 1:** Selected geometric parameters (Å, °)

Al—O1	1.881 (2)	Al—Br2	2.3029 (9)
Al—C1	1.927 (2)	Al—Br1	2.3046 (10)
			
O1—Al—C1	110.42 (13)	O1—Al—Br1	101.52 (8)
O1—Al—Br2	101.60 (7)	C1—Al—Br1	116.44 (5)
C1—Al—Br2	114.90 (9)	Br2—Al—Br1	110.07 (4)

**Table 2:** Hydrogen-bond geometry (Å, °)

*D*—H⋯*A*	*D*—H	H⋯*A*	*D*⋯*A*	*D*—H⋯*A*
C2—H2*A*⋯Br1	0.99	2.98	3.481 (3)	113
C5—H5*B*⋯Br1^i^	0.98	3.10	3.871 (4)	136

Symmetry code: (i) -x+{\script{1\over 2}}, -y+{\script{3\over 2}}, -z+1.

**Table 3:** Experimental details

Crystal data	
Chemical formula	[Al_2_Br_4_(CH_2_)(C_4_H_10_O)_2_]
*M*_r_	535.86
Crystal system, space group	Monoclinic, *C*2/*c*
Temperature (K)	100
*a*, *b*, *c* (Å)	8.3872 (6), 12.1039 (6), 18.1504 (12)
β (°)	95.646 (3)
*V* (Å^3^)	1833.7 (2)
*Z*	4
Radiation type	Mo *K*α
μ (mm^−1^)	8.87
Crystal size (mm)	0.31 × 0.25 × 0.08

Data collection	
Diffractometer	Bruker APEXII CCD
Absorption correction	Multi-scan (*SADABS*; Sheldrick, 1996 ▸)
*T*_min_, *T*_max_	0.291, 0.747
No. of measured, independent and observed [*I* > 2σ(*I*)] reflections	10600, 2027, 1736
*R*_int_	0.073
(sin θ/λ)_max_ (Å^−1^)	0.641

Refinement	
*R*[*F* ^2^ > 2σ(*F* ^2^)], *wR*(*F* ^2^), *S*	0.031, 0.076, 1.07
No. of reflections	2027
No. of parameters	83
H-atom treatment	H atoms treated by a mixture of independent and constrained refinement
Δρ_max_, Δρ_min_ (e Å^−3^)	0.74, −0.73

Computer programs: *APEX2* (Bruker, 2005 ▸), *SAINT* (Bruker, 2002 ▸), SHELXT (Sheldrick 2015*a* ▸), *SHELXL2018/3* (Sheldrick, 2015*b* ▸), and *SHELXTL* (Sheldrick 2008 ▸).

## supplementary crystallographic information

### Crystal data

**Table:**

[Al_2_Br_4_(CH_2_)(C_4_H_10_O)_2_]	*F*(000) = 1032
*M**_r_* = 535.86	*D*_x_ = 1.941 Mg m^−^^3^
Monoclinic, *C*2/*c*	Mo *K*α radiation, λ = 0.71073 Å
*a* = 8.3872 (6) Å	Cell parameters from 4301 reflections
*b* = 12.1039 (6) Å	θ = 3.1–32.5°
*c* = 18.1504 (12) Å	µ = 8.87 mm^−^^1^
β = 95.646 (3)°	*T* = 100 K
*V* = 1833.7 (2) Å^3^	Plate, colorless
*Z* = 4	0.31 × 0.25 × 0.08 mm

### Data collection

**Table:**

Bruker APEXII CCD diffractometer	1736 reflections with *I* > 2σ(*I*)
φ and ω scans	*R*_int_ = 0.073
Absorption correction: multi-scan (SADABS; Sheldrick, 1996)	θ_max_ = 27.1°, θ_min_ = 3.0°
*T*_min_ = 0.291, *T*_max_ = 0.747	*h* = −9→10
10600 measured reflections	*k* = −15→15
2027 independent reflections	*l* = −23→23

### Refinement

**Table:**

Refinement on *F*^2^	Primary atom site location: structure-invariant direct methods
Least-squares matrix: full	Secondary atom site location: difference Fourier map
*R*[*F*^2^ > 2σ(*F*^2^)] = 0.031	Hydrogen site location: mixed
*wR*(*F*^2^) = 0.076	H atoms treated by a mixture of independent and constrained refinement
*S* = 1.07	*w* = 1/[σ^2^(*F*_o_^2^) + (0.0337*P*)^2^ + 0.0465*P*] where *P* = (*F*_o_^2^ + 2*F*_c_^2^)/3
2027 reflections	(Δ/σ)_max_ = 0.001
83 parameters	Δρ_max_ = 0.74 e Å^−^^3^
0 restraints	Δρ_min_ = −0.73 e Å^−^^3^

### Special details

**Table:**

Geometry. All esds (except the esd in the dihedral angle between two l.s. planes) are estimated using the full covariance matrix. The cell esds are taken into account individually in the estimation of esds in distances, angles and torsion angles; correlations between esds in cell parameters are only used when they are defined by crystal symmetry. An approximate (isotropic) treatment of cell esds is used for estimating esds involving l.s. planes.

### Fractional atomic coordinates and isotropic or equivalent isotropic displacement parameters (Å^2^)

**Table:**

	*x*	*y*	*z*	*U*_iso_*/*U*_eq_	
Al	0.11619 (11)	0.71423 (7)	0.32918 (6)	0.0103 (2)	
Br1	−0.02876 (4)	0.77240 (3)	0.42346 (2)	0.01923 (12)	
Br2	0.27035 (4)	0.85713 (3)	0.29158 (2)	0.02154 (12)	
O1	0.2720 (3)	0.62389 (16)	0.38074 (13)	0.0112 (4)	
C1	0.000000	0.6327 (3)	0.250000	0.0131 (9)	
H1	−0.066 (4)	0.589 (3)	0.272 (2)	0.016*	
C2	0.2436 (4)	0.5550 (2)	0.44484 (19)	0.0150 (7)	
H2A	0.204255	0.601947	0.483872	0.018*	
H2B	0.345754	0.520776	0.465274	0.018*	
C3	0.1224 (4)	0.4655 (3)	0.4234 (2)	0.0202 (8)	
H3A	0.111673	0.417489	0.466116	0.030*	
H3B	0.158243	0.421669	0.382748	0.030*	
H3C	0.018501	0.499297	0.407455	0.030*	
C4	0.4055 (4)	0.5842 (3)	0.34074 (19)	0.0151 (7)	
H4A	0.388925	0.608372	0.288425	0.018*	
H4B	0.408161	0.502482	0.341600	0.018*	
C5	0.5618 (4)	0.6288 (3)	0.3758 (2)	0.0201 (7)	
H5A	0.650079	0.596834	0.351275	0.030*	
H5B	0.574490	0.609271	0.428443	0.030*	
H5C	0.563012	0.709361	0.370553	0.030*	

### Atomic displacement parameters (Å^2^)

**Table:**

	*U*^11^	*U*^22^	*U*^33^	*U*^12^	*U*^13^	*U*^23^
Al	0.0122 (5)	0.0062 (4)	0.0126 (5)	−0.0002 (3)	0.0013 (4)	0.0007 (3)
Br1	0.0223 (2)	0.01703 (18)	0.0192 (2)	0.00542 (12)	0.00634 (15)	−0.00335 (13)
Br2	0.0235 (2)	0.01272 (17)	0.0279 (2)	−0.00778 (12)	−0.00023 (15)	0.00757 (13)
O1	0.0110 (11)	0.0102 (10)	0.0127 (12)	0.0012 (8)	0.0025 (9)	0.0028 (8)
C1	0.016 (2)	0.0088 (19)	0.014 (2)	0.000	0.0011 (19)	0.000
C2	0.0167 (17)	0.0151 (15)	0.0128 (17)	0.0009 (12)	−0.0001 (13)	0.0045 (13)
C3	0.0199 (18)	0.0159 (16)	0.025 (2)	−0.0023 (13)	0.0051 (15)	0.0042 (14)
C4	0.0163 (17)	0.0143 (14)	0.0157 (17)	0.0016 (12)	0.0064 (14)	−0.0030 (13)
C5	0.0147 (18)	0.0257 (17)	0.0206 (19)	0.0015 (13)	0.0059 (14)	−0.0021 (14)

### Geometric parameters (Å, º)

**Table:**

Al—O1	1.881 (2)	C2—H2B	0.9900
Al—C1	1.927 (2)	C3—H3A	0.9800
Al—Br2	2.3029 (9)	C3—H3B	0.9800
Al—Br1	2.3046 (10)	C3—H3C	0.9800
O1—C2	1.470 (4)	C4—C5	1.500 (5)
O1—C4	1.473 (4)	C4—H4A	0.9900
C1—H1	0.89 (4)	C4—H4B	0.9900
C1—H1^i^	0.89 (4)	C5—H5A	0.9800
C2—C3	1.510 (4)	C5—H5B	0.9800
C2—H2A	0.9900	C5—H5C	0.9800
			
O1—Al—C1	110.42 (13)	H2A—C2—H2B	108.0
O1—Al—Br2	101.60 (7)	C2—C3—H3A	109.5
C1—Al—Br2	114.90 (9)	C2—C3—H3B	109.5
O1—Al—Br1	101.52 (8)	H3A—C3—H3B	109.5
C1—Al—Br1	116.44 (5)	C2—C3—H3C	109.5
Br2—Al—Br1	110.07 (4)	H3A—C3—H3C	109.5
C2—O1—C4	113.3 (2)	H3B—C3—H3C	109.5
C2—O1—Al	124.38 (19)	O1—C4—C5	110.4 (3)
C4—O1—Al	117.9 (2)	O1—C4—H4A	109.6
Al—C1—Al^i^	118.4 (2)	C5—C4—H4A	109.6
Al—C1—H1	105 (2)	O1—C4—H4B	109.6
Al^i^—C1—H1	111 (2)	C5—C4—H4B	109.6
Al—C1—H1^i^	111 (2)	H4A—C4—H4B	108.1
Al^i^—C1—H1^i^	105 (2)	C4—C5—H5A	109.5
H1—C1—H1^i^	107 (5)	C4—C5—H5B	109.5
O1—C2—C3	111.1 (3)	H5A—C5—H5B	109.5
O1—C2—H2A	109.4	C4—C5—H5C	109.5
C3—C2—H2A	109.4	H5A—C5—H5C	109.5
O1—C2—H2B	109.4	H5B—C5—H5C	109.5
C3—C2—H2B	109.4		
			
C1—Al—O1—C2	−89.5 (2)	Br1—Al—O1—C4	−170.52 (18)
Br2—Al—O1—C2	148.1 (2)	C4—O1—C2—C3	−91.5 (3)
Br1—Al—O1—C2	34.6 (2)	Al—O1—C2—C3	64.4 (3)
C1—Al—O1—C4	65.4 (2)	C2—O1—C4—C5	−85.6 (3)
Br2—Al—O1—C4	−57.0 (2)	Al—O1—C4—C5	116.9 (3)

Symmetry code: (i) −*x*, *y*, −*z*+1/2.

### Hydrogen-bond geometry (Å, º)

**Table:**

*D*—H···*A*	*D*—H	H···*A*	*D*···*A*	*D*—H···*A*
C2—H2*A*···Br1	0.99	2.98	3.481 (3)	113
C5—H5*B*···Br1^ii^	0.98	3.10	3.871 (4)	136

Symmetry code: (ii) −*x*+1/2, −*y*+3/2, −*z*+1.
